# Scientific Evaluation of Medicinal Plants Used for the Treatment of Cervicitis (Qorohe- Rahem) in Iranian Traditional Medicine

**DOI:** 10.22037/ijpr.2019.1100852

**Published:** 2019

**Authors:** Razieh Nabimeybodi, Rahele Zareshahi, Mojgan Tansaz, Marzieh Vahid Dastjerdi, Homa Hajimehdipoor

**Affiliations:** a *Department of Traditional Medicine, School of Traditional Medicine, Shahid Beheshti University of Medical Sciences, Tehran, Iran.*; b *Department of Persian Medicine, The School of Persian Medicine, Shahid Sadoughi University of Medical Sciences, Ardakan, Yazd, Iran*; c *Department of Pharmacognosy, Faculty of Pharmacy, Shahid Sadoughi University of Medical Sciences, Yazd, Iran.*; d *Department of Obstetrics and Gynecology, Tehran University of Medical Sciences, Tehran, Iran. *; e *Department of Traditional Pharmacy, School of Traditional Medicine, Traditional Medicine and Materia Medica Research Center, Shahid Beheshti University of Medical Sciences, Tehran, Iran.*

**Keywords:** Cervicitis, Qoruh-e- Rahem, Iranian Traditional Medicine, Persian medicine, Medicinal plants, Anti-inflammatory, Antibacterial, Antifungal, Wound healing

## Abstract

Cervicitis is an inflammatory condition of the cervix associated with upper genital tract infection and reproductive complications. Treatment for cervicitis in conventional system is the use of antibiotics and antifungal therapies and surgical interventions, but none of these treatments provides the definite efficacy in spite of high cost and side effect. So there is a need for an alternate therapy which is safe, effective, easily available and free from side effects. This review focuses on medicinal plants mentioned in main Iranian Traditional Medicine reference books. Medicinal plants mentioned in Iranian Traditional Medicine for treatment of Cervicitis were elicited and searched in electronic databases including Pub Med, Scopus, Science direct and Google Scholar to find studies that confirmed their efficacy. The findings included 31 plants belonging to 21 families. Research findings showed that the plants mentioned in Iranian Traditional Medicine resources can contribute to the recovery and treatment of cervicitis through anti- inflammatory, antioxidant, antibacterial and anti- fungal, wound healing and analgesic effects. Finding the medicinal plants effective on cervicitis based on ITM could suggest a better strategy for relieving and management of cervicitis symptoms especially in recurrent or persistent condition.

## Introduction

Cervicitis is a syndrome of cervical inflammation (1, 2) which is clinically defined as the presence of either mucopurulent discharge or cervical friability (easily bleeding induced by gentle passage of a swab through the Endocervical os) (1-6). More subtle signs of cervicitis include edema of the cervical ectropion (edematous ectopy) and vaginal pain (1, 3, 4, 6). Sacral backache, lower abdominal pain, and dyspareunia are other symptoms of cervicitis (4). Microscopic definitions involving the use of gram stain of cervical secretions includes either more than 10 white blood cells (WBCs) or more than 30 WBCs per high-power field (1, 3, 4, 6). Also, there is an evidence that it is asymptomatic in many cases (1, 2, 4). The incidence of cervicitis is as high as 30–45% in some sexually transmitted infections (STI) among clinic populations (4).

Cervicitis is most often caused by infection (1). Pathogens of *Chlamydia trachmatis* and *Neisseria gonorrhea* are the usual culprits (2-4). Less commonly, *Mycoplasma genitalium*, *Mycoplasma hominies*, *Ureaplasma urealyticum*, *Trichomona vaginalis,* and *Herpes simplex Virus* are implicated (2, 4). The causes of the rest of the cases remain unknown (3, 4, 7). However, in a few cases it may be attributed to chemical exposure or a foreign body such as a pessary (a device inserted into the vagina to support the uterus), cervical cap (a birth control device), or diaphragm. The condition may also be caused by an allergy to contraceptive spermicides or to latex in condoms (1).

Cervicitis can progress to Pelvic Inflammatory Disease (PID), ectopic pregnancy, chronic pelvic pain, tuboovarian abscess, damage and adhesion of tubal mucosa, and ultimately infertility (4, 5, 8) even in asymptomatic cases (2).

The initial therapy for cervicitis in conventional system is the use of antibiotics and antifungal therapies either orally and topically (4) leading to an imbalance in gut flora due to prolonged use of antibiotics (8). As a side effect, the increased drug resistance has reduced the therapeutic efficacy (4, 8, 9).

The failure of medical treatment (after two or three attempts) needs further surgical interventions by diathermy, cauterization, cryotherapy, and laser ablation which may cause further complications. None of these treatments not only provides a definite efficacy in spite of their relatively high cost, but also can lead to various adverse events. Due to the nature of these wide-ranging adverse effects, it is important to find effective therapies for genital infections which can be safer, more effective, easily available, and minimal adverse effects.

Complementary and Alternative Medicine (CAM) is progressively accepted and has been interested by the western mainstream medical community because of its less invasive, safer, effective, economical, and convenient therapies. The popularity of CAM has gradually increased, over the last few decades (8). Persian medicine (Iranian traditional medicine (ITM)) with thousands of years of history and hundreds of ancient texts is one of the oldest and richest alternative medicines.

Based on ITM literature, cervicitis is known as ″Qoruh- e- Rahem″, and characterized by pain and mucopurulent or bloody discharge. Qoruh is plural of Qarhah (Qorhah) which means wound in muscle tissue of the uterus and cervix. It can be developed by external causes such as bumps and falls (Sagtah) or internal causes such as dystocia, and flow of caustic humor (Insibab Khilt Hadd-e-Marari) to the uterus (10-14).

In ITM, Qarhah of the cervix can be diagnosed by inspection of cervix and observing the wound with endocervical discharge. Complications of Qoruh-e- Rahem include infertility and adhesion (10-14).

From ITM perspective, the treatment of cervicitis is a package of interventions, including lifestyle modification, medicinal plant therapies with different pharmacological therapeutic effects, and nondrug techniques such as massage (Dalk) and reflex therapy (Ghamz) used individually or in combination with each other.

The aim of the present study was to review the medicinal plants claimed to be effective on cervicitis based on the Iranian traditional medicine manuscripts that may be used as complementary and/or alternative to conventional treatments, based on the classical medicine, to find out more effective and safer treatment strategies.

## Experimental

This study is investigated medicinal plants which were used for the treatment of cervicitis/ Qoruhe-e-Rahem with keywords of Qoruh-e-Rahem or Qarhah (Qorhah) in the Iranian Traditional Medicine literature. The herbs were searched and extracted from 7 main traditional medicine reference books including *Liber Continent* (Al-Havi ) of Rhazes (*Abubakr Muhammad ibn Zakariyya al-Razi*, 8^th^ century AD), Canon of Medicine (Qanun fi al-*Teb)* of Avicenna (*Ibn Sina,*10^th^ and 11th centuries AD) (10), Great Elixir (*Exir Azam*) of Azam Khan (10^th^ century AD) (11), Treasure of the Khwarazm Shah (Zakhireh Kharazmshahi) of Jorjani (*Seyyed* *Ismaeil Jorjani*, 12^th^century AD) (12), Tohfat-ol-Momenin by Hakim Momen Husseini (17^th^ century AD) (15), Storehouse of Medicaments (*Makhzan-ol-Advieh*) of Aghili Khorasani (18^th^ century AD) (16) and Sharh-ol- Asbab of Nafis-ibn- Evas-e Kermani (15^th^ century AD) (17) as well as other manuscripts written at various times during 10^th^- 18^th ^centuries in both Arabic or Persian languages. Medicinal plants were categorized in Mofradat (simple ingredient) and Qarabadin (multi-component ingredient) (10, 11). Then, simple herbal drugs (Mofradat) were selected from extracted treatments 

To find matches for old names in modern scientific classification, two botany references (16, 18-21) and electronic databases as well as the plantlist.org suggested by the research team were used. Moreover, the opinions of distinguished scholars of ITM were also taken into consideration (Table 1).

**Figure 1:**
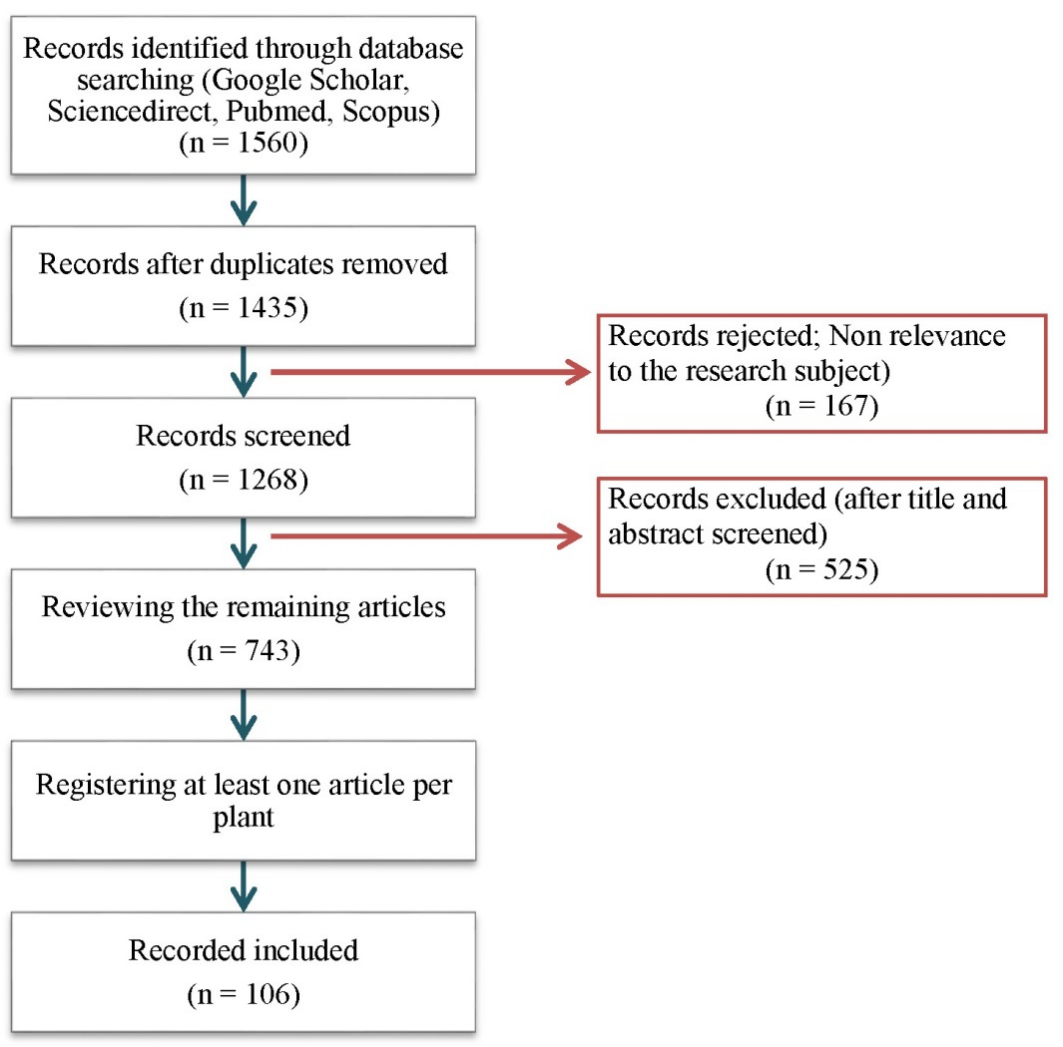
Flow diagram of the study selection

To investigate the pharmacological properties of the medicinal plants, electronic databases including Pub Med, Scopus, Google Scholar, and Sciencedirect were explored for each of these herbs. All retrieved articles which demonstrate the direct efficacy of these medicinal herbs or their mechanisms involved in cervicitis alleviation including anti-inflammatory, antioxidant, antimicrobial, antibacterial, antifungal, wound healing and analgesic effects were carefully considered in this study. Bibliography in the electronic databases covered all articles published between years 2000 to 2016. The search terms were ‘‘cervicitis’’, ‘‘cervicitis, uterine’’, ‘‘uterine cervicitis’’, ‘‘cervicitides’’, ‘‘cervicitides, uterine’’ and ‘‘uterine cervicitides’’ in title and abstract as well as the name of each herb in the whole text (Figure 1).

## Results and Discussion

After searching for plants effective against cervicitis (Qorohe-Rahem) in the 7 main Iranian Traditional Medicine texts, we reached 31 plants from 21 different families. Table 1, displays the medicinal plants used for the management of cervicitis in Iranian Traditional Medicine and all evidence confirming their efficacy are described individually in Table 2, 3 and 4.

The medicinal plants were categorized into 2 groups; 1) Mofradat which included simple ingredient herbal medicine, 2) Qarabadin which included multi component ingredient herbal medicines (containing 2 or more bioactive pharmaceutical substances) (10, 11, 22). Some routes of administrations for ITM drugs namely oral and topical included intravaginal administration (vaginal suppositories (Ferzajeh/farzaje, Sheiaf, Homoul, Zarour/Zarur), vaginal lavage/enema (Hoghneh)), external therapy (lotion (Tela), balm (Marham), cleansing (Estenja), steaming washing therapy and sitz bath (Abzan)) and rectal administration (retention enema and rectal infusion with liquid herbal medicine) (8, 22),

Ferzajeh and Homoul are two kinds of vaginal suppositories made of components that are kneaded and get dried in shade. Abzan is a traditional remedial sitz bath that is effective in treating gynecology disorders. In this procedure, the patient should sit in a tub filled with water in which a special plant is boiled before. Tela is a kind of lotion used topically. It is used on lower abdominal surface on uterine, pubic, external genitalia, and lumbosacral regions. The other form of drug administration is balm, which is used topically and is named Zemad in Iranian Traditional Medicine, containing some components and suitable liquid part, which makes it pasty. It needs to be dressed with a soft cloth. Cleansing with watery topical preparations is named Estenja (14, 22).

*Myrtus communis*

Leaves and fruits from *Myrtus communis* have been claimed to be effective for the management of cervicitis in different references of ITM. Analgesic (24), Antimicrobial (antibacterial, antifungal and antiviral) and antioxidant properties of compounds produced by *M. communis* have been reported in numerous studies. 1,8-cineole, linalool, eugenol, terpineol and terpinene as myrtle essential oils components have a good antibacterial effects against some gram positive and gram negative bacteria (25).

As demonstrated in several studies, the antioxidant capacity of plant extracts is strongly related to phenolic content. 

This activity is not a property of a single phenolic compound, but it is widely attributed to different phenolic phytochemical constituents. Particularly, anthocyanins, flavonoids and phenolic acids seem to be responsible for the antioxidant capacity of *Myrtus communis* (25). Rossi *et al.* in their study revealed that *M. communis* exerts potent anti-inflammatory effects *in-vivo* and offers a novel therapeutic approach for the management of acute inflammation (26). The effects of the essential oil and methanolic extract of *Myrtus communis* on *Trichomonas vaginalis* have been shown in Abdollahy *et al*. study (27).

*Juniperus Sabina*

*J. Sabina *is from Cupressaceae family. Emami *et al*. have reported antioxidant activity of leaves and fruits of *J. Sabina *(abhal). Antimicrobial activity of this plant has been confirmed (28, 29).

*Peucedanum officinale*

Many phytochemical investigations on this genus have confirmed *Peucedanum* species are rich in essential oils and coumarins. Our review confirmed that some *Peucedanum* species could have therapeutic effects of anti-inflammatory (30), antioxidant, and antimicrobial (31) .

*Commiphora opobalsamum*

*C. opobalsamum* is a small tree (5 m in height) that is found in abundance and widespread on mountains around the holy places such as Makkah Al-Mukarama, Al-Madina Al-Munawara (Al-hijaz area, KSA), and Al-Quds (Palestine). In addition, it is native to other areas such as Oman, Yemen, and Somaliland. Anti- inflammatory, antioxidant, and analgesic effects of *C*. *opobalsamum* have been reported by Al-salami *et al.* (32).

*Hyoscyamus sp.*

*Hyoscyamus niger* of Solanaceae family, commonly known as henbane, is widely distributed in Asia and Europe. The pharmacological evaluation of methanolic extract of the seeds of *H. niger* showed that it possesses potent analgesic and anti-inflammatory activities. The major chemical components, *e.g.* coumarinolignans specifically cleomiscosin, which is present in the seeds of *H. niger* is involved in the anti-inflammatory activity of methanolic extract of the seeds (33).

*Artemisia vulgaris*

Antimicrobial, antioxidant, and anti-inflammatory effects of this plant have been reported in the previous studies (34-36).

*Polyporus officinalis*

*Laricifomes officinalis* (*Polyporus officinalis*) is a wood-rotting fungus that grows on different hosts such as conifers. The mushroom is native to Europe, Asia, and North America. It has been used since the ancient times to treat sciatica, weakness of muscles, bronchitis, constipation, stomach and uterus pain, jaundice, fever, and insect bites. It also has diuretic and emmenagogue effects. The biological effects of *L. officinalis* including anti-viral (especially against smallpox, H5N1 influenza, and hepatitis C virus), anti-tuberculosis, anticoagulant immunomodulatory, and relieving dysmenorrhea, hemorrhoids, cough, and rheumatoid arthritis were confirmed for this fungus by studies performed in the recent decades (37).

*Lowsonia inermis*

Inhibitory action of henna against both gram negative and gram positive bacteria was proven (38). Chemical components of *L. inermis* have good antioxidant capacities and this species could be used as a potential source of new natural antioxidants (39).

*Trigonella foenum-graecum*

Leaves and seeds from *Trigonella foenum-graecum *has been claimed to be effective for management of cervicitis in different references of ITM. The anti-inflammatory, antioxidant, antimicrobial, analgesic, and wound healing effects of *T. foenum-graecum *have been reported (40-43). The presence of saponins and flavonoids as the major compounds in* T. foenum-graecum* may explain the anti- inflammatory activity of this plant (44). 

*Prangos ferulaceae*

Antioxidant effect of *P. ferulaceae* was reported by Coruh *et al*. (45). 

*Apium graveolens*

Anti-inflammatory, antimicrobial and antioxidant effects of *A. graveolens* have been reported. Baananou reported essential oil and extracts of *A. graveolens* aerial parts have antiulcerogenic and antibacterial activities (46-48).

*Malva sylvestris*

In animal models, *M. sylvestris* presented antinociceptive effects and anti-inflammatory action (49, 50) in mucous membranes and in carrageenan-induced paw edema when applied topically. The antioxidant and radical scavenger properties of this herb (*in-vitro*) were presented by a study of Della Greca *et al*. *M. sylvestris* is effective as an anti-inflammatory agent when used locally in the skin (50). Antimicrobial effects of this plant also have been reported by Gasparetto *et al*. (49).

One study evaluated the effect of *M. sylvestris* topical cream on burn wound healing in the rats. (51).

*Descurainia sophia*

Anti-inflammatory, antioxidant, and analgesic activities of *Descurainia sophia* (flixweed) were attributed to the presence of phenolic compounds (52).

*Allium porrum*

Antimicrobial and antioxidant effects of *A. porrum* were reported by Mnayer* et al. *(53).

*Boswellia carteri*

The antimicrobial activity of the essential oil of *B. carteri* was individually evaluated against different microorganisms including fungi, gram-positive, and gram-negative bacteria strains (54). In one study *B. carteri* was used as a mixture with three plants for diabetic wound healing. The results of this study revealed this treatment is a promising method for wound healing in diabetic mice (55).

Anti-inflammatory, antifungal, antioxidant activities, and analgesic effects of this plant have been reported (56, 57).

*Fraxinus excelsior*

Antioxidant, antibacterial, and anti-inflammatory activities of* F. excelsior* were shown in the previous reports (58, 59). 

*Plantago major*

The main caffeic acid derivative in *P. major* is plantamajoside having anti-inflammatory, antioxidant, and antibacterial activities (60). The wound-healing properties of* P. major* were evaluated using an ex-vivo porcine wound healing model. Ethanol and water extracts stimulated wound healing in porcine skin (61).

*Phaseolus vulgaris*

Antimicrobial and antioxidant activity of *Ph. Vulgaris *have been reported (62-64).

*Pistacia lentiscus*

Resin of *P. lentiscus* showed 100% inhibition of inflammation at 800 mg/kg i.p. injection, without any toxicity in mice (65). *P. lentiscus* virgin fatty oil promotes significantly wound contraction and reduces epithelization period in rabbit model (66). Antifungal and antioxidant effects also have been reported (67).

*Commiphora myrha*

Pharmacological studies showed that myrrh exhibited analgesic, anti-inflammatory, and antimicrobial activities (57, 68). The analgesic activity of *Commiphora *extract and pure compounds supported the use of myrrh for wound and pain in indigenous medicines (69).

*Trachyspermum copticum*

*Trachyspermum copticum*, an annual plant which grows in Iran, has white flowers and small fruits. Some biological effects of the fruits of *T. copticum* such as antiviral, anti-inflammatory, antifungal, analgesic, antinociceptive, and antioxidant activity have been confirmed (70-72).

*Narcissus tazetta*

Ethanol extracts of aerial parts of this plant showed antimicrobial effects. Aerial parts of *N. tazetta* have flavonoids, terpenoids, and alkaloids (73, 74).

*Verbena Officinalis*

Casanova *et al*. reported 50% methanolic extract and caffeoyl derivatives could be potentially considered as excellent and readily available sources of natural antifungal and antioxidant compounds (75). A topical preparation containing at least 3% of *V. officinalis *methanolic extract showed an anti-inflammatory and analgesic effects (76). 

*Foeniculum vulgare*

Antioxidant activity of *F .vulgare* was demonstrated by various studies. The fruits have shown antioxidant activity in animal models. In a study investigating antioxidant properties of different parts of *F. vulgare*, the shoots had the highest radical scavenging and lipid peroxidation inhibiting activity. Despite of these positive reports, the results of a study on different fractions of fruit and their major chemical compounds did not show strong antioxidant activities from isolated *F. vulgare *components (77).

Oral administration of a methanol extract of *F. vulgare* fruits exhibited inhibitory effects against acute and subacute inflammatory diseases and showed a central analgesic activity in rat and mice (77).

*F. vulgare* essential oil possesses a strong antifungal activity against different fungal species (78) and aqueous extract of *F. vulgare* has showed potent antibacterial activity (79).

*Cydonia oblonga*

Quince is a tree cultivated as a medicinal plant in the Middle East, South Africa, and Central Europe. One study indicates that the mucilage obtained from quince seeds accelerates wound healing in rabbits (80). Polyphenolic compounds of *C. oblonga* were responsible for the antimicrobial effects of this plant (81). 

Branca *et al*. reported the antioxidant activities of quince. Evaluation of the antioxidant activity of methanolic extract of C. *oblonga* showed that its peel extract has the highest antioxidant capacity. The half maximal inhibitory concentration (IC_50_ values) of quince pulp, peel, and jam extracts were correlated with total content of caffeoylquinic acids (82). Essafi-Benkhadir *et al. *reported anti-inflammatory effect of polyphenolic extract of the *C. blonga* (83). 

*Anethum graveolens*

Many studies were done about *A. graveolens*. In these studies antibacterial, antifungal, and antioxidant activity of *A. graveolens*has have been confirmed (84-86). 

*Ficus carica*

Antimicrobial, antioxidant and anti-inflammatory effects of different dosage forms prepared from different parts/extracts of the *F.carica* are well documented (87, 88).

*Rosa damescena*

In Shohayeb *et al. *study, essential oil and different extracts of petals of the *R. damascena* were evaluated for their antimicrobial activities against three gram- positive and seven gram- negative bacterias, one acid-fast bacterium and three fungi. Rose oil and all tested rose fractions indicated broad spectrum antibacterial activity against all tested bacteria and fungi (89).

The *R. damascena* similar to many aromatic and medicinal plants exhibits antioxidant properties (41). Hajhashemi *et al*. showed *R. damascena* contains active analgesic ingredients acting both centrally and peripherally. Results of one study showed hydroalcoholic extract of this plant relieved inflammatory pain in an animal model (90).

*Crocus sativus (Saffron)*

*Crocus sativus *have many pharmacological properties such as anti-inflammatory, antioxidant, antimicrobial, and wound healing. Extract of pollen of this herb has therapeutic effects on wounds induced by mustard in animals. Chemical constituents of saffron are crocins, crocetin, picrocrocin, β-carotene, and safranal. These compounds have antioxidant activity (91).

*Aristolochia longa*

Antifungal and antimicrobial effects of this plant have been reported. Aristolochic acids isolated from this plant show antimicrobial activity against some microorganisms (76, 92).

*Olea europaea*

Anti-inflammatory, antioxidant, and antimicrobial activities of different parts of *O. europaea* have been demonstrated in different studies (93-96).

Pereira *et al.* has reported that low concentrations of olive leaves extract has combined antibacterial and antifungal actions (97). For the first time Koca *et al*. in 2011 reported wound healing activity of the aqueous extract of *O. Europea *leaves. Antioxidant and antimicrobial effects of olive leaves might explain their beneficial effects on wound healing (98).

**Figure 2:**
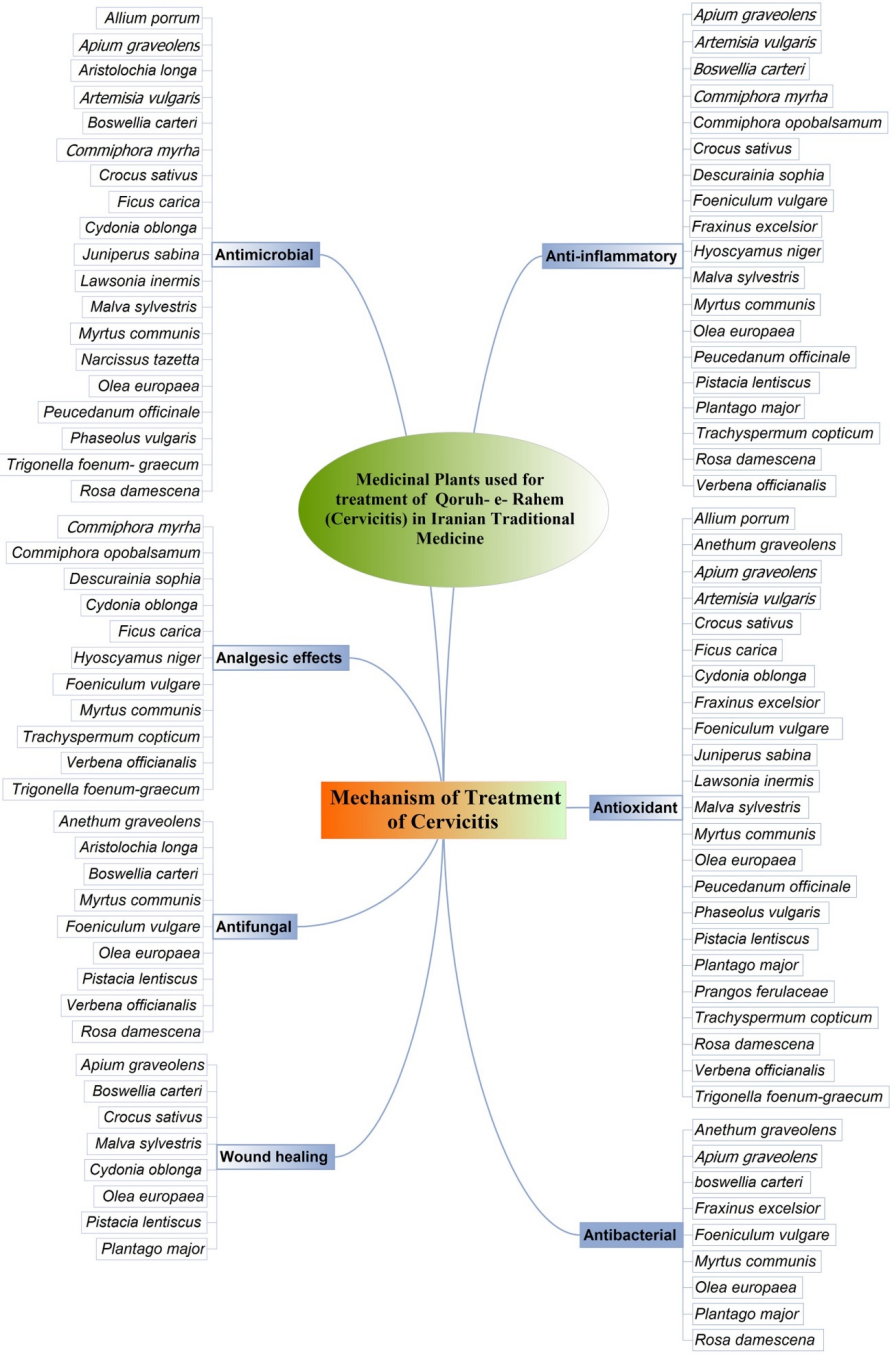
Mechanism of action for medicinal plant

**Table 2:** Studies on anti-inflammatory activities of plants used for treatment of cervicitis cited in ITM references

	**Scientific name**	**Part/extract**	**Active constituent**	**Reference**
1.	*Allium porrum*	-----	--------	----
*2.*	*Anethum graveolens*	Essential oil	Sabinen	(84)
3.	*Apium graveolens*	Ethanolic extract of seeds	-----	(48)
4.	*Aristolochia longa Aristolochia rotunda*	-----	------	---
5.	*Artemisia vulgaris*	Hydroalcoholic extract	Rutin, hydroxybenzoic acid and caffeic	(35)
6.	*Boswellia carteri*	Aquouse extract	----	(57)
7.	*Commiphora myrha*	Aqeouse extract of resin	sesquiterpene,diterpenen, triterpenic acids	(57)
8.	*Commiphora opobalsamum*	Petrolatum ether, Chloroform, extract of aerial parts	Triterpens, flavonols, mearnsetin, quersetin, ascorbic acid	(32)
9.	*Crocus sativus*	Methanolic extract of aerial parts	-----	(24)
10.	*Cydonia oblonga*	Polyphenol ectract	polyphenols	(52)
11.	*Descurainia sophia*	Petrolatom extract of seeds	Coumarins	(83)
12.	*Ficus carica*	---	----	---
13.	*Foeniculum vulgare*	Methanolic extract of fruites	----	(77)
14.	*Fraxinus excelsior*	Ethanolic extract of the bark	coumarins	(59)
15.	*Hyoscyamus niger Hyoscyamus albus Hyoscyamus sp.*	Methanolic extract of seeds	coumarinolignans	(33)
16.	*Juniperus sabina*	---	----	---
17.	*Lawsonia inermis*	---	lawsochylin A, lawsonaphthoate	(99)
18.	*Malva sylvestris*	Ethanolic extract of leaves	malvidin3- glucoside,scopoletin,quercetin	(50)
19.	*Myrtus communis*	----	----	(25)
20.	*Narcissus tazetta*	----	----	--
21.	*Olea europaea*	Chloroformic and methanolic extract of leaves	Iridoids, Flavonoids	(100)
22.	*Peucedanum officinale*	----	----	(30)
23.	*Phaseolus vulgaris*	---	---	---
24.	*Pistacia lentiscus*	Resin	Flavonoids	(65)
25.	*Plantago major*	Ethanolic and aquase extract of leaves	Phenolic compounds, Polysaccharides and polyphenolic compounds	(60, 61)
26.	*Polyporus officinalis*	---	---	----
27.	*Prangos ferulaceae*	----	-----	----
28.	*Rosa damescena*	Ethanolic extract of air dried and petals	Phenolic compounds	(90)
29.	*Trachyspermum copticum*	Ethanolic extract of seeds	Flavonoids and glycosides	(72)
30.	*Trigonellafoenum-graecum*	Methanolic extract of seeds	Glycoside and steroidal moieties	(44)
31.	*Verbena officianalis Verbena *sp.	Methanolic extract of leaves	Iridoids and caffeoyl derivatives	(75)

**Table 3:** Studies on antimicrobial activities of plants used for treatment of cervicitis cited in ITM references

	**Scientific name**	**Part/extract**			**Active constituent**		**Activities**	**Reference**	
1.	*Allium porrum*	Essential oil			Dipropyl disulfide, dipropyltrisulfide, methyl propyl disulfide, polyphenols		Antibacterial,	(53)	
*2.*	*Anethum graveolens*	Ethanolic extract of flowers			proanthocyanidins		Antifungal, antibactrial	(85, 86)	
3.	*Apium graveolens*	Essential oil			----		Antibacterial	(47)	
4.	*Aristolochia longa Aristolochia rotunda*	Aqueous extract of root, Hexane and benzene extract of dried root			Flavonoides,Saponins/ Aristolochic acid and aristolactam		Antifungal, Antibacterial	(76)	
5.	*Artemisia vulgaris*	Ethanolic Extract of aerial parts			Flavonoids, phenols		Antimicrobial,	(34)	
6.	*Boswellia carteri*	Essential oils			limonene		Antibacterial, antifungal	(54)	
7.	*Commiphora myrha*	Ethanol and ether extracts			-----		Antimicrobial	(69, 101)	
8.	*Commiphora opobalsamum*	Petrolatum ether			Triterpens, flavonols		Antimicrobial	(102)	
9.	*Crocus sativus*	Methanolic extract of aerial parts			---		Antimicrobial	(103)	
10.	*Cydonia oblonga*	Fruit aqueous acetone extract			chlorogenicacid		Antimicrobial	(104)	
11.	*Descurainia sophia*	----			----		-----	----	
12.	*Ficus carica*	Methanol extract			---		Antibactrial Antifungal	(87)	
13.	*Foeniculum vulgare*	Essential oil, Aquase extract			Anethol		Antimicrobial	(78)	
14.	*Fraxinus excelsior*	dichloromethane of leaves			----		Antibacterial	(105)	
15.	*Hyoscyamus niger Hyoscyamus albus Hyoscyamus sp.*	Ethanolic extract of shoot and root			Alkaloids		Antimicrobial	(106)	
16.	*Juniperus sabina*	Essential oil of fruit			Flavonoids		Antimicrobial	(28)	
17.	*Lawsonia inermis*	----			----		Antimicrobial	(38)	
18.	*Malva sylvestris*	Methanolic extract of aerial parts			----		Antibactrial	(104)	
19.	*Myrtus communis*	Essential oil, Ethanolic and methanolic and ethylacetat extract of leaves and berries, Ethanolic extract			Polyphenolic compounds, phenolic acids, tannins, flavonoids		Antimicrobial	(25)	
20.	*Narcissus tazetta*	Ethanol extract of aerial parts			---		Antimicrobial	(74)	
21.	*Olea europaea*	Aqueous extracts of leaves			Phenolic compounds		Antifungal and antibacterial	(97)	
22.	*Peucedanum officinale*	----			----		Antimicrobial	(31)	
23.	*Phaseolus vulgaris*	Acetonic extract			Tannins		Antibacterial	(64)	
24.	*Pistacia lentiscus*	Mastic			----		Antimicrobial	(67)	
25.	*Plantago major*	Ethanolic and aquase extract of leaves			Phenolic compounds, Polysaccharides and polyphenolic compounds		Antibacterial	(60)	
	**Scientific name**		**Part/extract**	**Active constituent**		**Activities**		**Reference**
26.	*Polyporus officinalis*		----	----		----		----
27.	*Prangos ferulaceae*		Fruit extract	limonene, α-pinen, and humulene		Antibacterial		(45)
28.	*Rosa damescena*		Essential oil and ethanolic extract	----		Antibacterial and Antifungal		(107)
29.	*Trachyspermum copticum*		Essential oil	Phenols		Antimicrobial		(71)
30.	*Trigonellafoenum- graecum*		Ethanolic extract of mucilage	---		Antibacterial		(102)
31.	*Verbena officianalis Verbena *sp.		Methanolic extract of leaves	Flavonoids and caffeoyl derivatives		Antimicrobial		(75)

**Table 4:** Studies on antioxidant activities of plants used for treatment of Cervicitis cited in ITM references

		**Scientific name**	**Part/extract**			**Active constituent**		**Reference**	
1.		*Allium porrum*	Essential oil			Dipropyl disulfide, dipropyltrisulfide, methyl propyl disulfide, polyphenols		(53)	
*2.*		*Anethum graveolens*	Essential oil			Tannins, Flavonoid, Alkaloids		(86)	
3.		*Apium graveolens*	Methanolic and ethanolic extrac of seeds			flavonoids, phenols		(108)	
4.		*Aristolochia longa Aristolochia rotunda*	-------			-------		--	
5.		*Artemisia vulgaris*	Ethanolic Extract of aerial parts			Flavonoids, phenols		(34)	
6.		*Boswellia carteri*	Aquouse extract			----		(56)	
7.		*Commiphora myrha*	Ethanol and ether extracts			----		(69)	
8.		*Commiphora opobalsamum*	Petrolatum ether, Chloroform, extract of aerial parts			Triterpens, flavonols, mearnsetin, quersetin, ascorbic acid		(102)	
9.		*Crocus sativus*	Methanolic extract of aerial parts			----		(103)	
10.		*Cydonia oblonga*	Methanolic extract of pulp and peel and seed and jam			Phenolic compounds		(82)	
11.		*Descurainia sophia*	Ethanolic extract of seeds			Phenols		(109)	
12.		*Ficus carica*	----			Polyphenols, Flavonoids, Anthocyanins		(87)	
13.		*Foeniculum vulgare*	Ethanolic extract of fruits			----		(77)	
14.		*Fraxinus excelsior*	Ethanolic extract of leaves			Fraxetin, esculetin		(59)	
15.		*Hyoscyamus niger Hyoscyamus albus Hyoscyamus sp.*	----			------			
16.		*Juniperus sabina*	Methanolic extract of Leaves and fruits			----		(29)	
17.		*Lawsonia inermis*	Ethanolic extract of leaves			----		(39)	
18.		*Malva sylvestris*	---			----		(104)	
19.		*Myrtus communis*	Ethanolic extracts of berries			Flavonoids, Tannins, α-tocopherol		(25)	
20.		*Narcissus tazetta*	---			---		---	
21.		*Olea europaea*	leaf extract			Flavonols, Flavans-3-ols, Flavones		(93)	
22.		*Peucedanum officinale*	----			----		(31)	
23.		*Phaseolus vulgaris*	Methanolic extract of bean			Phenols		(62)	
24.		*Pistacia lentiscus*	Resin			Flavonoids		(67)	
25.		*Plantago major*	Ethanolic and aquase extract of leaves			Phenolic compounds, Polysaccharides and polyphenolic compounds		(60)	
26.		*Polyporus officinalis*	---			---		---	
27.		*Prangos ferulaceae*	---			Coumarines, Alkaloids, Flavonoids, and Terpenoids		(45)	
28.		*Rosa damescena*	Hydroalcohlic and ethanolic extract, fresh flower, spent flower),Essential oil			Phenolic compounds		(41)	
29.	*Trachyspermum copticum*			Ethanolic extract of seeds	Terpenoids and Flavonoids		(70)	
30.	*Trigonellafoenum-graecum*			Ethanolic extract of mucilage	Galactomannan		(110)	
31.	*Verbena officianalis Verbena *sp.			Methanolic extract of leaves	Flavonoids and Caffeoyl derivatives		(75)	

## Conclusion

Overall, we found 31 plants from 21 different families cited in the Iranian Traditional Medicine as therapies for cervicitis. Most of these plants had been shown *in-vit*ro and/or *in-vivo* anti-inflammatory, antimicrobial and antioxidant effects (Figure 2).

Treatment with medicinal plants has attracted the attention of scientists and there are throwbacks to the traditional medicine in many countries (111). Medicinal plants used in traditional medicine and ethnopharmacology may be valuable sources for discovering new therapies (112). In Iranian Traditional Medicine manuscripts, various medicinal plants with different pharmacological activities have been introduced. In this article, the medicinal plants claimed to be effective in cervicitis have been collected from Canon of Medicine, Makhzan-Al-advieh, Tohfeh-Al-Momenin, Exire Azam and Sharh-ol- Asbab, Zakhireh Kharazmshahi and their possible efficacy and pharmacodynamics in modern medicine were surveyed. Some of them such as *Cydonia oblonga*, *Olea europaea*, *Ficus carica,* and *Allium porrum* have nutritional value and are routinely used in human diets.

Cervicitis, presenting as inflammation of the uterine cervix, is a syndrome usually caused by infection (113). Oxidative stress, microbial infections, and inflammation are associated with human uterine cervicitis (4, 7, 113). Different mechanisms of action could be considered for these medicinal plants including anti-inflammatory and antimicrobial properties, analgesic, antioxidant activity, and wound healing activities. Finding the medicinal plants effective on cervicitis based on ITM could suggest a better strategy for relieving and management of cervicitis symptoms especially in recurrent or persistent conditions.

It should be contemplated that even though exploring ITM literature may lead to the identification of effective natural medicines for the management of different ailments such as cervicitis; however, confirming clinical trials or supportive high-quality observational studies needs to be accomplished before routine administration of herbal medicines or treatment regimens (Tadabeer) recommended in traditional medicine texts to affirm efficacy and safety of these treatments.
